# Amelioration of the brain structural connectivity is accompanied with changes of gut microbiota in a tuberous sclerosis complex mouse model

**DOI:** 10.1038/s41398-024-02752-y

**Published:** 2024-01-31

**Authors:** Christine Chin-jung Hsieh, Yu-Chun Lo, Hsin-Hui Wang, Hsin-Ying Shen, You-Yin Chen, Yi-Chao Lee

**Affiliations:** 1 Biomedical Translation Research Center, Academia Sinica, Taipei, Taiwan; 2 Ph.D. Program in Medical Neuroscience, College of Medical Science and Technology, Taipei Medical University, Taipei, Taiwan; 3 Neuroscience Research Center, Taipei Medical University, Taipei, Taiwan; 4 Department of Biomedical Engineering, National Yang Ming Chiao Tung University, Taipei, Taiwan; 5 International Master Program in Medical Neuroscience, College of Medical Science and Technology, Taipei Medical University, Taipei, Taiwan

**Keywords:** Neuroscience, Physiology

## Abstract

Tuberous sclerosis complex (TSC) is a genetic disease that causes benign tumors and dysfunctions in many organs, including the brain. Aside from the brain malformations, many individuals with TSC exhibit neuropsychiatric symptoms. Among these symptoms, autism spectrum disorder (ASD) is one of the most common co-morbidities, affecting up to 60% of the population. Past neuroimaging studies strongly suggested that the impairments in brain connectivity contribute to ASD, whether or not TSC-related. Specifically, the tract-based diffusion tensor imaging (DTI) analysis provides information on the fiber integrity and has been used to study the neuropathological changes in the white matter of TSC patients with ASD symptoms. In our previous study, curcumin, a diet-derived mTOR inhibitor has been shown to effectively mitigate learning and memory deficits and anxiety-like behavior in *Tsc2*^+/−^ mice via inhibiting astroglial proliferation. Recently, gut microbiota, which is greatly influenced by the diet, has been considered to play an important role in regulating several components of the central nervous system, including glial functions. In this study, we showed that the abnormal social behavior in the *Tsc2*^+/−^ mice can be ameliorated by the dietary curcumin treatment. Second, using tract-based DTI analysis, we found that the *Tsc2*^+/−^ mice exhibited altered fractional anisotropy, axial and radial diffusivities of axonal bundles connecting the prefrontal cortex, nucleus accumbens, hypothalamus, and amygdala, indicating a decreased brain network. Third, the dietary curcumin treatment improved the DTI metrics, in accordance with changes in the gut microbiota composition. At the bacterial phylum level, we showed that the abundances of Actinobacteria, Verrucomicrobia, and Tenericutes were significantly correlated with the DTI metrics FA, AD, and RD, respectively. Finally, we revealed that the expression of myelin-associated proteins, myelin bassic protein (MBP) and proteolipid protein (PLP) was increased after the treatment. Overall, we showed a strong correlation between structural connectivity alterations and social behavioral deficits, as well as the diet-dependent changes in gut microbiota composition.

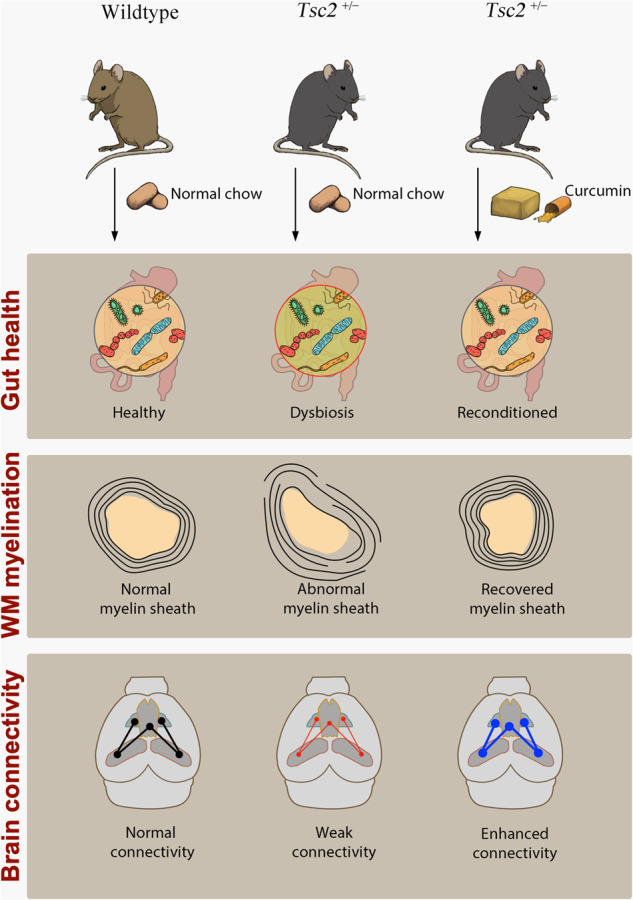

## Introduction

Tuberous sclerosis complex (TSC) is an autosomal dominant genetic disease characterized by benign tumor formation throughout the whole body, including the central nervous system. More than 80% of the people with TSC manifest neurological complications such as early-onset epilepsy and other neuropsychiatric symptoms such as autism spectrum disorder (ASD), intellectual disability, which are collectively known as TSC-associated neuropsychiatric disorders (TANDs) [1]. Among these symptoms, individuals with TSC are at especially high risk for ASD. It is estimated to be from 16 to 61% of the TSC population that shows features of ASD, compared to about 2% risk in the general population [2].

TSC results from a single gene mutation of either the *TSC1* or *TSC2* gene, which encodes the proteins hamartin or tuberin, respectively. These two proteins form a heterodimer functional unit that inhibits mammalian/mechanistic target of rapamycin (mTOR) complex signaling [3]. Since mTOR and its associated proteins are key regulators during brain development and control the synaptic network, the aberrations in mTOR caused from the loss of *TSC1* or *TSC2* will therefore result in synaptic and neuroanatomical abnormalities [3–5].

Several lines of evidence demonstrated that, in addition to neuronal abnormalities, glial dysfunction also plays a crucial role in the synaptic pathogenesis of TSC. In the brain of TSC patients, mTOR hyperactivity promotes aberrant astroglial proliferation, hypomyelination, and as well as abnormal growth of giant cells, which represent a cell type with features of immature macroglial or neuronal lineage cells [6]. Among these glial pathological hallmarks, white matter (WM) deficits and/or myelin abnormalities have become one of the prominent clinical phenotypes in TSC and are consistently observed in both TSC patients and several animal models [7–10].

The development and maintenance of an intact WM are achieved via proper oligodendroglial development. Oligodendroglia or oligodendrocytes (OLs) undergo a complex process, including proliferation, migration, and differentiation in a timely manner, to generate multilamellar myelin sheath, which insulates the axons to propagate electrical signals [11]. Thus, the intactness of myelin is important for communications between neurons and brain regions, and eventually connecting the whole brain, establishing an extensive brain network. Indeed, myelin damage, either demyelination or hypomyelination, causes cognitive impairments [12] and neuropsychiatric diseases, including autism spectrum disorder (ASD) [13].

Over the past decades, diffusion tensor imaging (DTI), or diffusion magnetic resonance imaging (dMRI) tractography, has been utilized extensively to monitor the neural circuitry through observing WM changes in a variety of neurodegenerative diseases and neurodevelopmental disorders, including ASD and TSC (see review papers [14, 15]). The tractography technique can assess the connectivity strength between grey matter (GM) regions by computing measures from the diffusion tensor model [16]. The measures derived from the tensor model include fractional anisotropy (FA), axial, radial, and mean diffusivities (AD, RD, and MD, respectively) [17]. In general, FA gives the overall microstructural integrity and tells the degree of coherency of the tissue structure. MD shows the average water diffusivity within the tissue, providing information on the cellularity, such as edema, or necrosis [18]. Both AD and RD have been used widely to characterize WM changes and related pathologies. AD reflects more of axonal integrity, whereas RD is more sensitive to myelin integrity [19–21].

Based on these different attributes given by the DTI metrics, DTI has been a useful tool for detecting the subtle WM alterations in the normal-appearing white matter (NAWM) [13, 22–24]. For example, a decreased FA value in NAWM of Alzheimer’s disease and ASD has been reported when compared to the healthy controls, suggesting that FA is a positive scalar that correlates with the connectivity ‘strength’. Likewise, a reduction in FA is also observed in the NAWM of TSC in general [25–27], as compared to the controls, as well as in TSC with ASD when compared to the controls or TSC without ASD [28, 29]. In addition, the FA value can be reversed upon mTOR inhibition in TSC [30], suggesting that the abnormal WM originated from the genetic defect of TSC could be modified pharmacologically, even after birth, and can be detected by the DTI modality.

WM development after birth progresses quite dynamically and can be modified by environmental factors, including diets and intestinal microbial composition in early life [31]. Gut microbiota has been increasingly considered a key component of the developing gut-brain axis (GBA) through many different pathways and mechanisms [32]. One of the mechanisms is myelination regulation, which contributes to WM development after birth. In recent years, a number of studies have consistently demonstrated that prefrontal cortical (PFC) myelination is greatly impacted by gut dysbiosis [33–35]. The PFC region is a key brain region that is involved in several neuropsychiatric disorders, including cognitive impairment and ASD, both of which patients exhibit gut dysbiosis [33, 36, 37]. Thus, much effort has been put into seeking the possibility of treating ASD through altering the gut microbiota composition with dietary pre- and probiotic administration [38].

Our previous study has shown that the dietary solid lipid curcumin particle (SLCP) treatment can ameliorate cognitive deficits in *Tsc2*^*+/−*^ mice, accompanied with the reversal of the regional FA values in gray matter (GM) regions, as well as the enrichment of the cortical myelination [39]. To further probe into the therapeutic mechanisms of SLCP treatment, we herein proposed that SLCP treatment also alters the WM structures through beneficially changing gut microbiota composition in *Tsc2*^*+/−*^ mice at the adolescent age. We further explored the myelination at the prefrontal cortex (PFC) region of the brain using immunostaining and electron microscopy to further confirm that the developing microbiota-gut-brain (MGB) axis can be re-established by oral administration of SLCP.

## Materials and methods

### Animals

The animal protocols used in this study followed the guidelines and regulations of the Taipei Medical University Institutional Animal Care and Use Committee (IACUC). All experiments were performed in accordance with the US Public Health Service Policy on Humane Care and Use of Laboratory Animals. All animals were housed in an air-conditioned vivarium with free access to food and water and a 10/14 h light/dark cycle. The male *Tsc2*^*+/−*^ heterozygous knockout mice (JAX stock #004686; B6;129S4-*Tsc2*^<tm1Djk>^/J) aged 6-week-old to 14-week-old mice were used in this study, which were purchased from the Jackson Laboratory (Bar Harbor, ME). Previous reports have indicated that this mouse model shows cognitive deficits and autistic-like behavior without the presence of seizures and brain malformations such as cortical tubers, subependymal nodules, and subependymal giant cell astrocytoma [40, 41].

### Experimental design

**Fig. 1:**
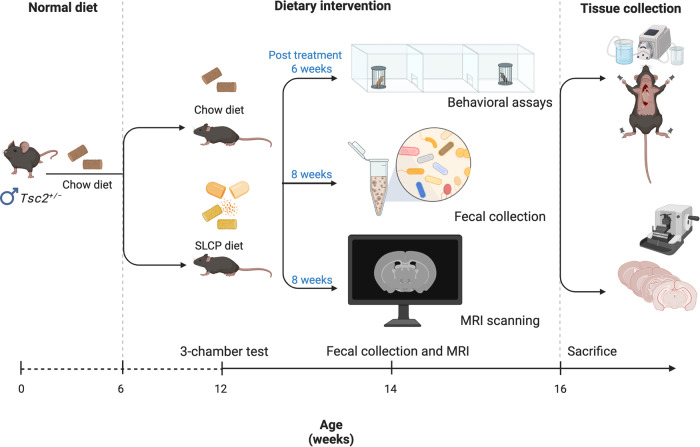
Experimental timeline. At 6 weeks of age, the male *Tsc2*^*+/−*^ mice started the 0.2% SLCP diet. At 12–14 weeks of age, WT (*N* = 9), *Tsc2*^*+/−*^ (*N* = 8), and *Tsc2*^*+/−*^/SLCP (*N* = 8) mice were assessed with a 3-chamber social preference test and social novelty test. At 12–14 weeks of age, the animals underwent MRI acquisition and fecal collection. At 16 weeks of age, mice were sacrificed with transcardial perfusion; brain slices were obtained for subsequent histological and immunostaining analyses.

The experimental design and timeline are illustrated in Fig. 1. At 4 weeks of age, after weaning, male mice were randomly divided into three experimental groups: first, wildtype mice fed with normal diet chow (WT, *N* = 9), second, *Tsc2*^*+/−*^ mice fed with normal diet chow (*Tsc2*^*+/−*^, *N* = 8), and third, *Tsc2*^*+/−*^ mice fed with SLCP-containing chow (*Tsc2*^*+/−*^/SLCP, *N* = 8). The pups were assigned and allocated evenly per litter. The sample size was determined based on our previous study [39]. Beginning at 6 weeks of age, the *Tsc2*^*+/−*^/SLCP group started the 0.2% SLCP diet. After 6–8 weeks of treatment, approximately at 12–14 weeks of age, WT, *Tsc2*^*+/−*^, and *Tsc2*^*+/−*^/SLCP mice were assessed with social behavioral tests: 3-chambered social preference test and social novelty test. At 12–14 weeks of age, the animals underwent MRI acquisition and fecal collection. Subsequently, mice were sacrificed and perfused; brain slices were obtained for further histological and immunostaining analyses. The investigators who performed behavioral tests, MRI, and gut microbiome analysis were blinded to the groups.

### Solid lipid curcumin particle (SLCP) treatment

The treatment was administered orally. Longvida® solid lipid curcumin particles (SLCP, Verdure Sciences Inc., Noblesville, IN, USA) were purchased from Da Yi Biotech & Health Food Co., Ltd. (Chiayi City, Taiwan). Each capsule contains 400 mg curcumin formulation containing ~80 mg patented lipid-coated SLCP. The curcumin powder was thoroughly mixed into standard rodent chow after being pulverized (Oriental Yeast Co. Ltd, Tokyo, Japan) and 3% hydroxypropyl methylcellulose (Wei Ming Pharmaceutical, Taipei, Taiwan) to yield a concentration of 0.2% SLCP. The mixture was blended with a small quantity of autoclaved water, molded into pellets, and dried in an oven set at 65 °C. One gram of curcumin-containing chow contained 2 mg of SLCP powder. Since the average daily food intake for a mouse is estimated to be 0.19 g/g body weight/day according to the reference [42], the estimated daily intake (dosage) of SLCP is to be 0.38 mg/g body weight for a mouse.

### Behavioral tests

The 3-chambered social interaction test is divided into two parts of tests. The first test is the social preference test, which consists of three phases. Each phase is a 10-minute session. Phase I: Habituate subject mice to the center chamber. During this phase, the doorways to the left and right chambers are closed. Phase II: Habituate mice to all three chambers. During this phase, the doorways to the left and right chambers were open, allowing subject mice to explore all three chambers. Phase III: Test for sociability. During this phase, the novel animal was introduced to one of the side chambers, with a container restraining the movements of the novel animal. In the other chamber, a container, with the same appearance, is placed without the novel animal. Then the doorways were open, allowing subject mice to choose which chamber they preferred to explore.

The second part is to test for social novelty. An additional phase IV is followed by phase III during the social preference test. Phase IV: Test for preference for social novelty. During this 10-min session, a stranger animal with the container was introduced to replace the empty container. Then the doorways were opened, allowing subject mice to choose between the familiar mouse and the stranger mouse. The time spent in the side chambers was calculated.

The social preference index was calculated as the difference between the time spent in the chamber with the novel animal (T_animal_) and the novel object (T_object_) in relation to the total time spent in both chambers ((T_animal_ − T_object_)/(T_animal_ + T_object_)). The social novelty index was calculated as the difference between the time spent in the chamber containing the stranger animal (T_stranger_) and the familiar animal (T_familiar_) in relation to the total time spent in both chambers ((T_stranger_ − T_familiar_)/(T_stranger_ + T_familiar_)) [43]. The indices are expressed as ratio of the total time spent in the chambers.

### MRI acquisition

MR images were acquired using a 7 Tesla scanner with a 30 cm diameter bore (Bruker Biospec 70/30 USR, Ettlingen, Germany), and the linear volume coil was used to transmit the radio frequency pulses. For the radio frequency signal retrieval, a planar surface coil (T7399V3; Bruker Corp., Billerica, MA, USA) was placed over each mouse’s head. During each MRI session, the mouse was anesthetized by inhalation of 3% isoflurane (Attane™ Isoflurane, Minrad Inc., NY, USA), in combination with 20% O_2,_ 75% N_2_, and 5% CO_2_. Magnetic field homogeneity was optimized using the fast automated shimming technique by mapping along projections (FASTMAP) with first-order shims on an isotropic voxel of 7 × 7 × 7 mm^3^ encompassing the imaging slices. Turbo spin echo (TSE) T2 images were acquired TR = 2500 ms, TE = 33 ms, matrix size = 256 × 256 × 15, field of view (FOV) = 20 × 20 mm^2^, voxel size = 0.08 × 0.08 × 0.4 mm^3^, slice thickness = 0.4 mm, 15 horizontal slices. Diffusion images were acquired using the DtiEpi SpinEcho sequence, TR = 3750 ms and TE = 40.28 ms, matrix size = 50 × 50 × 15 pixels, FOV = 20 × 20 mm^2^, slice thickness = 0.4 mm, 15 horizontal slices, the diffusion time was 20 ms. The diffusion encoding duration was 6 ms. A total of 12 diffusion sampling directions were acquired.

### Diffusion tensor imaging tractography analysis

Preprocessing steps and DTI analyses were performed by DSI studio software (http://dsi-studio.labsolver.org) including the motion correction, mask set up, and reconstruction according to the instructions provided. A model-based DTI reconstruction method, based on details provided by Jiang et. al. [44], was used to construct the diffusion map. First, the Allen mouse brain atlas and ROIs [45] were first reconstructed according to the same dimensions as the diffusion images using MATLAB (ver. R2021, Mathworks, Inc., Natick, MA, USA). The reference atlas was then loaded to co-register with the diffusion images. Tract-based analysis was performed for areas associated with social behavior, which are the prefrontal cortex (PFC), nucleus accumbens (NAc), hypothalamus (HyTh), amygdala (Amyg), and the hippocampus (Hipp), which is further segmented into Cornu Amonis 1–3 and dentate gyrus (CA1, CA2, CA3, and DG), to obtain the diffusion indices, FA, MD, AD and RD values.

### Brain slice preparations and immunostaining

Animal transcardial perfusion was performed and then they were sacrificed and decapitated. After being extracted from the skull, the brains were fixed with 4% PFA in PBS, pH 7.4 at 4 °C for 24 h. Fixed brains were then dehydrated with 30% sucrose/PB and underwent frozen sectioning. Sections with 20 μm-thickness were obtained for subsequent histological analysis. For immunofluorescence staining, antigen retrieval was done according to the procedures described in the Abcam protocol (Abcam, Cambridge, MA, USA). Briefly, the brain slices were immersed in sodium citrate buffer (10 mM, with 0.05% Tween 20, pH = 6) at 95 to 100 °C for 20 to 25 min. After cooling, the subsequent immunostaining procedures were carried out. Myelin basic protein (MBP) (808401, BioLegends, San Diego, CA, USA), and myelin proteolipid protein (PLP) (ab105784, Abcam, Cambridge, MA, USA) were used to determine the expression levels of myelin-associated proteins.

### Transmission electron microscopy image analysis

Mice were subject to transcardial perfusion with perfusion fixative buffer and then decapitated. After being extracted from the skull, the brains underwent post-fixation with 2% PFA/2.5% glutaraldehyde in 0.1 M Cacodylate, pH 7.4 at 4 °C for 24 h. The brain tissues were then washed with 0.1 M Cacodylate/7% sucrose and post-fixed with 1% OsO4 for 1–2 h. Then wash again with 0.1 M Cacodylate/7% sucrose, followed by dehydration steps, 70%, 80%, 90%, 95%, and 100% for 15–45 min. The tissues were then put in propylenoxide for 10 min 3 times and Propylenoxide/EPON was mixed with a 1:1 ratio overnight. Embedding and hardening at 62 °C for 3 days were then followed by sectioning. Electron micrographs were obtained using HT7700 (HITACHI, Tokyo, Japan). For g-ratio calculations, Fiji was used to define the diameters of axons and axons with myelin sheath. The formula is as follows:

$$G=\frac{d}{D}$$

where d = diameter length of the axon, D = diameter length of the axon with myelin. The myelin thickness is determined by the surface area of the axon including myelin minus the surface area of the axon.

### Collection of fecal samples and extraction of DNA

Stools from the WT, *Tsc2*^*+/−*^, and *Tsc2*^*+/−*^/SLCP groups were collected before the MRI scanning when the animals were 12 weeks old and SLCP-treated for 6 weeks. The fresh fecal samples were collected in sterile plastic tubes and stored at −80 °C immediately before DNA extraction. Total bacterial DNA in fecal samples was extracted using the QIAmp Fast DNA Stool Mini Kit (QIAGEN, Hilden, German) according to the manufacturer’s manual. Isolated DNA was stored at −20 °C until subsequent gene sequencing.

### 16S rDNA gene sequencing

To analyze the gut microbiota, fecal samples of the animals were collected for 16 S rDNA sequencing. The gut microbiome sequencing was carried out by the Taipei Medical University Core Laboratory of Human Microbiome (Taipei, Taiwan). The universal primers 341 F (5ʹ-CCTACGGGNGGCWGCAG-3ʹ) and 805 R (5ʹ-GACTACHVGGGTATCTAATCC-3ʹ) with Illumina overhang adapter sequences in the forward (5ʹ-TCGTCGGCAGCGTCAGATGTGTATAAGAGACAG-3ʹ) and reverse (5ʹ-GTCTCGTGGGCTCGGAGATGTGTATAAGAGACAG-3ʹ) primers were used to amplify the V3-V4 highly variable region of the 16 S rDNA gene sequence. The 16 s rDNA was PCR-amplified by the Illumina Miseq platform (Illumina). The Nextera XT Index kit (Illumina Inc.) was used to adapt the Illumina sequencing adapters and dual-index barcodes to the targets in the amplicon and the quantity and qualify sequenced library were checked by QSep100 Analyzer (BiOptic Inc., Taiwan).

### Bioinformatic and statistical analysis of gut microbiome

The universal primers (341 F and 805 R) that were used to amplify the V3-V4 region of the bacterial 16 S rRNA genes were first removed from the demultiplexed, paired reads using cutadapt (v 1.12; DOI:10.14806/ej.17.1.200). The filtered reads were processed in the R environment (v 3.3.3) using R package DADA2 (v 1.3.5) [46] following the workflow described in Callahan et al. 2016 [47] without performing rarefying procedure. Briefly, the forward and reversed reads were filtered and trimmed based on the read quality score and read length. Dereplication was then performed to merge identical reads, and then reads were subjected to the denoise DADA2 algorithm which alternates between error-rate estimation and sample composition inference until they converge on a jointly consistent solution. Finally, the paired reads were merged requiring a minimum of 20 bp overlap and chimeras were subsequently removed. At this point, we obtained a list of V3-V4 sequence variants (SVs) found in our samples that were inferred by DADA2, as well as the frequency of each SV in each sample. Taxonomy assignment was performed using the SILVA database (v138) [48] as the reference with a minimum bootstrap confidence of 80. Multiple sequence alignment of the SVs was performed with DECIPHER (v2.2.0) and the phylogenetic tree was constructed from the alignment using phangorn (v2.2.0) [49]. The count table, taxonomy assignment results, and phylogenetic tree were consolidated into a phyloseq object, and community analyses were performed using phyloseq (v1.19.1) [50]. The alpha-diversity indices were calculated using the estimate_richness function from the phyloseq package. Statistical comparison between treatment and control was performed with the exact Wilcoxon-Mann-Whitney test (at α = 0.05). UniFrac distances were calculated using the GUniFrac package (v1.1) to assess the community dissimilarity between groups [51]. Principal coordinate analysis (PCoA) ordination on UniFrac distances was performed and the adonis and betadisper functions from the vegan package (v2.4; https://CRAN.R-project.org/package=vegan) were used to conduct statistical analysis for the dissimilarity of composition among groups and the homogeneity of dispersion respectively. Microbiota enrichment analysis between groups was carried out by using the Linear Discriminant Analysis (LDA) Effect Size (LEfSe) method with alpha set at 0.05 (Kruskal-Wallis and Wilcoxon tests) and logarithmic LDA score of 2 or more [52] and visualized as cladogram by using GraPhlAn [53]. Additional statistical analysis and relevant graphs were generated using the Microbiome Analyst website (https://www.microbiomeanalyst.ca/) [54].

### Statistical analysis

For determining the statistical significances among the three groups in behavioral tests, immunostaining image analyses, DTI metrics, one-way ANOVA with Tukey’s test as the *post-hoc* analysis for multiple comparisons was performed. A probability value of *p* < 0.05 was used to determine statistical significance. All data are presented as mean ± standard error of the mean (SEM). The above datasets were tested and confirmed for the normal distribution using D’Agostino & Pearson, Anderson-Darling, and Kolmogorov-Smirnov tests. Levene’s test was performed to test the homogeneity of variance and an equal variance was confirmed between the groups.

For TEM analysis, when the axon diameter was plotted against the myelin thickness or the g-ratio, the simple linear regression was performed and overall slopes and elevations (or intercepts) were tested. A probability value of *p* < 0.05 was used to determine statistical significance. For the myelin breakdown, the normality of the datasets was tested and confirmed using D’Agostino & Pearson, Anderson-Darling, Shapiro-Wilk, and Kolmogorov-Smirnov tests. Therefore, one-way ANOVA with *post-hoc* Tukey’s test was performed. A probability value of *p* < 0.05 was used to determine statistical significance. All data are presented as mean ± standard error of the mean (SEM).

For the relative abundance of gut microbiota datasets, two-tailed Mann–Whitney test was used to compare the WT and *Tsc2*^*+/−*^mice. For the comparisons among three groups, Kruskal Wallis with *post-hoc* uncorrected Dunn’s test for the multiple comparisons was performed. A probability value of *p* < 0.05 was used to determine statistical significance. All data are presented as mean ± standard error of the mean (SEM).

All the statistical analyses mentioned above were performed using Prism version 10 (GraphPad Software, Inc., San Diego, CA, USA).

## Results

### Social behavioral deficits of Tsc2^+/−^ mice can be rescued by dietary SLCP treatment

**Fig. 2:**
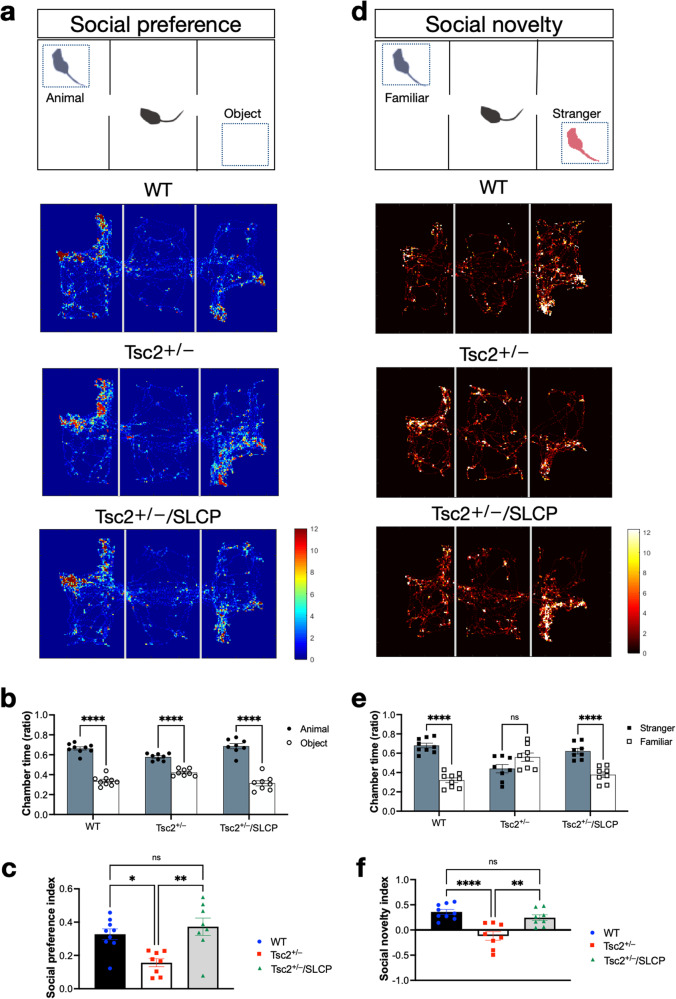
SLCP treatment ameliorated social behavioral deficits in *Tsc2*^*+/−*^ mice. **a** Three-chamber apparatus set-up of social behavioral preference test and the heatmaps of the representative mouse tracks from each group. **b** The time spent in the chambers of either the animal or the object was shown for each group. **c** The social preference indices between the object and the animal were shown. **d** Three-chamber apparatus set-up of social behavioral novelty test and the heatmaps of the representative mouse tracks from each group. **e** The time spent in the chambers of either the familiar animal or the stranger animal was shown for each group. **f** The social novelty indices between the familiar animal and the stranger animal were shown. WT (*N* = 9), *Tsc2*^*+/−*^ (*N* = 8), and *Tsc2*^*+/−*^/SLCP (*N* = 8) mice were assessed. Data represent the mean ± SEM. *p < 0.05, **p < 0.01, ***p < 0.001, ****p < 0.0001, n.s. not significant. One-way ANOVA, *post hoc* Tukey’s test.

To investigate whether SLCP treatment is able to improve the ASD-like phenotypes of *Tsc2*^*+/−*^mice, since ASD is common in TSC patients, we performed social behavioral tests. During the 3-chambered social test, all 3 groups spent significantly more time around the animal over the object, as indicated by the representative mouse tracks from each group (Fig. 2a) and the quantitative bar graph (Fig. 2b). The social preference indices (PI) between the object and the animal were calculated among the three groups, WT (0.305 ± 0.038), *Tsc2*^*+/−*^ (0.156 ± 0.025), and *Tsc2*^*+/−*^/SLCP (0.342 ± 0.046) at 12 weeks of age. The PI of the *Tsc2*^*+/−*^ mice was significantly lower than that of the WT mice (*p* = 0.048, one-way ANOVA test with *post-hoc* Tukey’s test), and the PI of the *Tsc2*^*+/−*^/SLCP group was higher than that of the *Tsc2*^*+/−*^ mice (*p* = 0.015, one-way ANOVA test with *post-hoc* Tukey’s test). This result demonstrated that *Tsc2*^*+/−*^ mice showed impaired social behavior, which was rescued by 8 weeks of SLCP treatment (Fig. 2c).

We also performed the social novelty preference test to assess the social memory and the novelty preference for each group (Fig. 2d). WT and *Tsc2*^*+/−*^/SLCP mice showed a tendency to spend increased time in the stranger animal chamber, as compared to the familiar animal chamber. *Tsc2*^*+/−*^ mice, however, showed a preference for the familiar animal or no preference for the stranger animal (Fig. 2e). The social novelty index (NI) between the stranger and the familiar animal was calculated among the three groups, WT (0.359 ± 0.049), *Tsc2*^*+/−*^ (−0.053 ± 0.096), and *Tsc2*^*+/−*^/SLCP (0.313 ± 0.070) at 12 weeks of age. The NI of the *Tsc2*^*+/−*^ mice was significantly lower than that of the WT mice (*p* = 0.002, one-way ANOVA test with *post-hoc* Tukey’s test), and the NI of the *Tsc2*^*+/−*^/SLCP group was higher than that of the *Tsc2*^*+/−*^ mice (*p* = 0.007, one-way ANOVA test with *post-hoc* Tukey’s test). The result demonstrated that *Tsc2*^*+/−*^ mice had a significantly lower novelty preference, and the dietary SLCP-treated group showed a reversal of this behavior. **(**Fig. 2f).

### Dietary SLCP treatment enhanced the structural connectivity in Tsc2^+/−^ mice

**Fig. 3:**
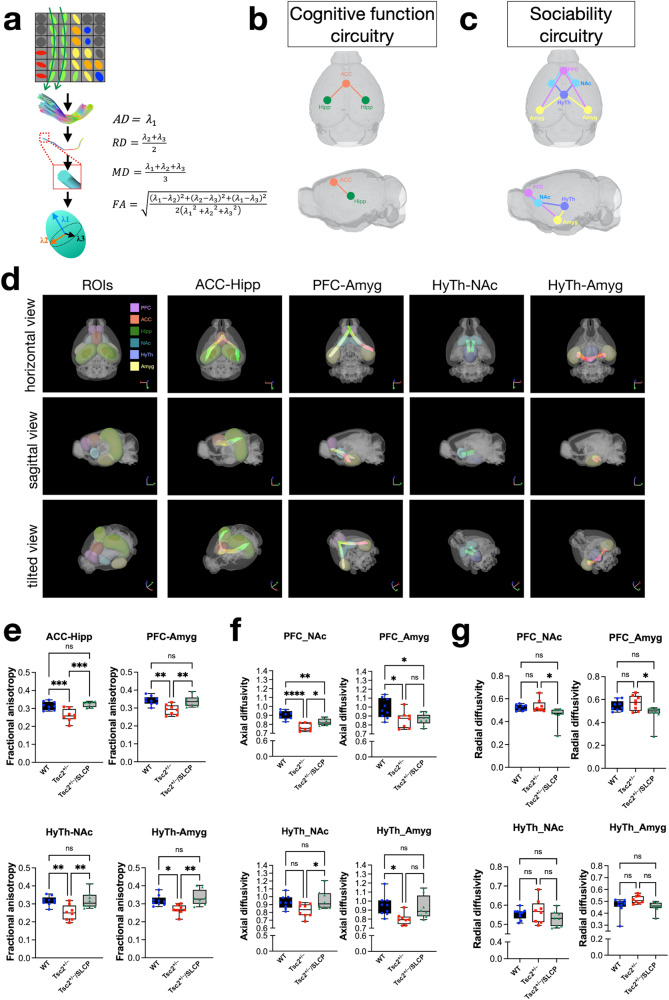
SLCP treatment enhanced structural connectivity in *Tsc2*^*+/−*^ mice. **a** The schematic diagram of the fiber tractography analysis and algorithms for each diffusion tensor metric. **b** The schematic diagram of connections within the cognitive function circuitry is shown. **c** The schematic diagram of connections within the sociability circuitry is shown. **d** The representative trajectories delineated by the software between the designated brain regions as indicated are shown. **e, f, g** FA, AD, and RD values of estimated fiber tracts connecting between indicated regions are shown. Abbreviations: ACC: anterior cingulate cortex; CA1: cornu ammonis 1; FA: fractional anisotropy; AD: axial diffusivity; RD: radial diffusivity. WT (*N* = 9), *Tsc2*^*+/−*^ (*N* = 8), and *Tsc2*^*+/−*^/SLCP (*N* = 8) mice were assessed. Data represent the mean ± SEM. *p < 0.05, **p < 0.01, n.s. not significant, One-way ANOVA test, post hoc Tukey’s test.

Since neurodevelopmental disorders such as ASD are often associated with WM defects [13, 24], we next aimed to investigate whether *Tsc2*^*+/−*^ mice exhibited weak connectivity between important brain regions involved in social behavior. We used a fiber tractography approach derived from the DTI algorithm to determine FA, MD, AD, and RD values (Fig. 3a). The tract-based DTI analysis delineates the fiber bundles connecting the regions of interest and to determine the connectivity strength within the circuitry. In our previous study, we found that *Tsc2*^*+/−*^ mice showed decreased cognitive function and decreased FA in the corpus callosum [39]. Therefore, we further analyzed the connectivity between the regions that are considered important in learning and memory function, the anterior cingulate cortex (ACC) and hippocampal CA1 [55, 56] (ACC-Hipp) (Fig. 3b). In this study, we showed that *Tsc2*^*+/−*^ mice exhibited abnormal social behavior, hence we further analyzed the connectivity in social behavior-related circuitry, which involves prefrontal cortex (PFC), nucleus accumbens (NAc), hypothalamus (HyTh), and amygdala (Amyg) [57, 58] (Fig. 3c).

The schematic fiber trajectories were estimated by DSI studio [59] and were shown in Fig. 3d. We analyzed the connecting fibers in between ACC-Hipp, PFC and Amyg (PFC-Amyg), HyTh and NAc (HyTh-NAc), and finally HyTh and Amyg (HyTh-Amyg). The FA value of fibers connecting between ACC-Hipp, PFC-Amyg, HyTh-NAc, and HyTh-Amyg in *Tsc2*^*+/−*^ mice was significantly decreased as compared to the WT and *Tsc2*^*+/−*^/SLCP (Fig. 3e). As for the AD value of the fiber in between PFC-NAc, PFC-Amyg, and HyTh-Amyg in *Tsc2*^*+/−*^ mice showed a significantly lower value as compared to the WT mice (Fig. 3f). We further analyzed the RD value and found that *Tsc2*^*+/−*^ mice did not significantly differ from the WT mice. However, *Tsc2*^*+/−*^/SLCP mice showed significantly decreased RD values as compared to the *Tsc2*^*+/−*^ mice in the fibers connecting the PFC-Amyg, as well as the fiber between PFC-NAc (Fig. 3g). We have also examined MD values for these connecting fibers, but found no significant differences in these brain regions (data not shown). In summary, our DTI tractography data indicated that the *Tsc2*^*+/−*^ mice have impaired structural connectivity in the cognition- and social interaction-related circuitry; as reflected by the FA and AD values. After the dietary treatment of SLCP, the FA and RD values were altered among these regions (Table 1).

**Table 1:** Summary table for DTI metrics and TEM analysis.

Parameters	Brain regions	WT	*Tsc2*^*+/−*^	*Tsc2*^*+/−*^*/*SLCP
FA	PFC-Amyg	0.342 ± 0.009	0.290 ± 0.010**	0.338 ± 0.011^##^
	PFC-NAc	0.322 ± 0.011	0.264 ± 0.010	0.308 ± 0.009
	HyTh-NAc	0.317 ± 0.009	0.251 ± 0.016	0.320 ± 0.017
	HyTh-Amyg	0.318 ± 0.010	0.268 ± 0.10	0.333 ± 0.016
AD	PFC-Amyg	0.986 ± 0.039	0.861 ± 0.035*	0.863 ± 0.025*
	PFC-NAc	0.905 ± 0.017	0.769 ± 0.016	0.826 ± 0.013
	HyTh-NAc	0.931 ± 0.029	0.825 ± 0.032	0.959 ± 0.047
	HyTh-Amyg	0.949 ± 0.041	0.801 ± 0.024	0.928 ± 0.049
RD	PFC-Amyg	0.544 ± 0.016	0.571 ± 0.024	0.477 ± 0.031^#^
	PFC-NAc	0.523 ± 0.009	0.536 ± 0.021	0.459 ± 0.029
	HyTh-NAc	0.555 ± 0.010	0.567 ± 0.023	0.530 ± 0.016
	HyTh-Amyg	0.463 ± 0.024	0.516 ± 0.013	0.455 ± 0.017
G-ratio slope	Anterior forceps	0.1019 – 0.1380	0.0719 – 0.1400***	0.1047 – 0.1707^##^
		Normal myelination	Abnormal myelination	Recovered myelination
Axon caliber	Anterior forceps	0.63 – 0.99	0.58 – 0.84	0.61 – 0.92
		Normal distribution	Shifted toward smaller caliber	Close to normal distribution

*p < 0.05, **p < 0.01, ***p < 0.001 when compared to the WT group.

# *p* < 0.05, ## *p* < 0.01 when compared to the *Tsc2*^*+/−*^group.

Data are represented as mean ± SEM.

Statistical significances were determined by one-way ANOVA test, post-hoc Tukey’s test for multiple comparisons.

### Tsc2^+/−^mice displayed alteration in diameter distributions of myelinated axons and abnormal myelin sheath lamination

**Fig. 4:**
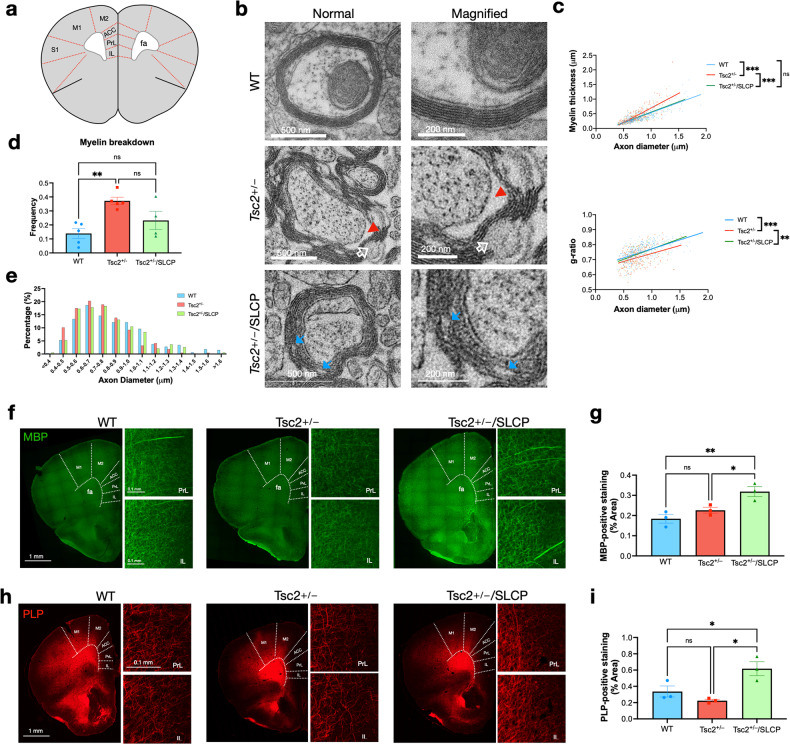
*Tsc2*^*+/−*^ mice showed abnormal myelin sheath and SLCP treatment induced MBP expression. **a** Schematic illustration of the white matter structure, anterior forceps, which was analyzed using TEM; and the gray matter structures, prefrontal cortical regions (PrL and IL), which were analyzed by MBP and PLP fluorescent immunostaining (adapted from Allen brain atlas). **b** Electron micrographs of axons in the body of anterior forceps for the three groups. *Upper panel*. The intact myelin sheath in the WT mice. *Middle panel*. Abnormal myelin sheath in the *Tsc2*^*+/−*^ mice. The red arrowhead indicates the space between the axon-myelin interface. The white open arrow indicates myelin sheath detachment. *Lower panel*. Myelin sheath with mild myelin splits in the *Tsc2*^*+/−*^/SLCP mice as indicated by the blue arrows. **c** *Upper panel*. The myelin thickness was plotted as the function of axon diameter. *Lower panel*. The g-ratio was plotted as the function of axon diameter. **d** The quantitative bar graph shows the number of axons with the myelin breakdown. **e** Quantitative analysis of myelinated axons with the diameter ranging from <0.4 to >1.6 μm as indicated. WT (*N* = 5), *Tsc2*^*+/−*^ (*N* = 5), and *Tsc2*^*+/−*^/SLCP (*N* = 4) mice were assessed. **f** A representative fluorescence immunostaining image from each group is shown for visualizing the immunoreactivity of MBP staining. **g** The quantitative bar graph shows the MBP-positive staining area (%) for the three groups. **h** A representative fluorescence immunostaining image from each group is shown for visualizing the immunoreactivity of PLP staining. **i** The quantitative bar graph shows the PLP-positive staining area (%) for the three groups. WT (N = 3), *Tsc2*^*+/−*^ (N = 3), and *Tsc2*^*+/−*^/SLCP (*N* = 3) mice were assessed. Abbreviations: TEM: transmission electron microscopy; MBP: myelin basic protein; PLP: myelin proteolipid protein; fa: anterior forceps; PrL: prelimbic cortex; IL: infralimbic cortex; ACC: anterior cingulate cortex; M1, M2: motor cortex 1 or 2, respectively. Data represent the mean ± SEM. *p < 0.05, **p < 0.01, ***p < 0.001, n.s. not significant, One-way ANOVA test, *post hoc* Tukey’s test.

To find out the factors that contribute to the decreased FA and AD in *Tsc2*^*+/−*^mice, we performed transmission electron microscopy analysis in the white matter structure anterior forceps (fa) of corpus callosum (Fig. 4a). This region is the white matter structure that is nearest to the PFC and it connects the anterior frontal lobes between two hemispheres [60, 61]. The *Tsc2*^*+/−*^ mice showed pronounced myelin sheath abnormalities and alteration in diameter distributions of myelinated axons as compared to the WT mice (Fig. 4b). The myelinated axons of the *Tsc2*^*+/−*^ mice showed axo-glial detachment, as indicated by the red arrowhead, and myelin splitting, as indicated by the white open arrow (Fig. 4b, middle panel). The dietary SLCP treatment improved the myelin sheath formation, as the myelin sheath showed to be more compacted (Fig. 4b, lower panel).

When we calculated the myelin thickness as the function of axon diameter, the *Tsc2*^*+/−*^ mice showed significantly increased myelin sheath thickness (Fig. 4c, upper panel). From the g-ratio plot, the *Tsc2*^*+/−*^ mice exhibited decreased g-ratio as compared to the WT mice, indicating an increased myelin sheath (Fig. 4c, lower panel). The increased myelin sheath surface area likely resulted from the spaces between the axo-glia interface and myelin splitting. The SLCP treatment group showed similar slopes to the WT mice. The *Tsc2*^*+/−*^ mice showed an increased frequency of myelin breakdown, whereas the SLCP-treated group showed a trend of lower frequency of myelin breakdown (Fig. 4d, Table 1**)**. The number of myelinated axons was profoundly increased for axons with a diameter of 0.4–0.9 μm in *Tsc2*^*+/−*^ mice when compared to the WT mice (Fig. 4e, Table 1).

### Myelination changes in the PFC of Tsc2^+/−^ mice after dietary SLCP treatment

Since myelin basic protein (MBP) is a major player in myelin sheath compaction [62], we performed MBP immunostaining for the three groups to assess the cortical and hippocampal myeloarchitecture. We examined the MBP-positive areas in the PFC, designated as prelimbic (PrL) and infralimbic (IL) cortex (Fig. 4f). As the results showed, there were no significant differences between the WT mice and the *Tsc2*^*+/−*^ mice. However, SLCP treatment significantly increased the immunoreactivity of MBP, as indicated by the MBP-positive staining area (Fig. 4g). We further analyzed the MBP expression in hippocampal region and found no significant differences between the groups (Supplementary data Fig. S1). We also probed into another myelin-associated protein, myelin proteolipid protein (PLP) in the PFC regions (Fig. 4h). We also further calculated the PLP-positive staining area and found that similar to MBP, *Tsc2*^*+/−*^/SLCP group showed increased immunoreactivity of PLP (Fig. 4i). Since PFC myelination has been recently demonstrated to be regulated through gut microbiota [33, 35], and gut bacteria have been shown to be influenced by the diet such as curcumin [63], we next analyzed the gut microbial composition in these mice.

### Gut microbiota composition significantly altered after dietary SLCP treatment

**Fig. 5:**
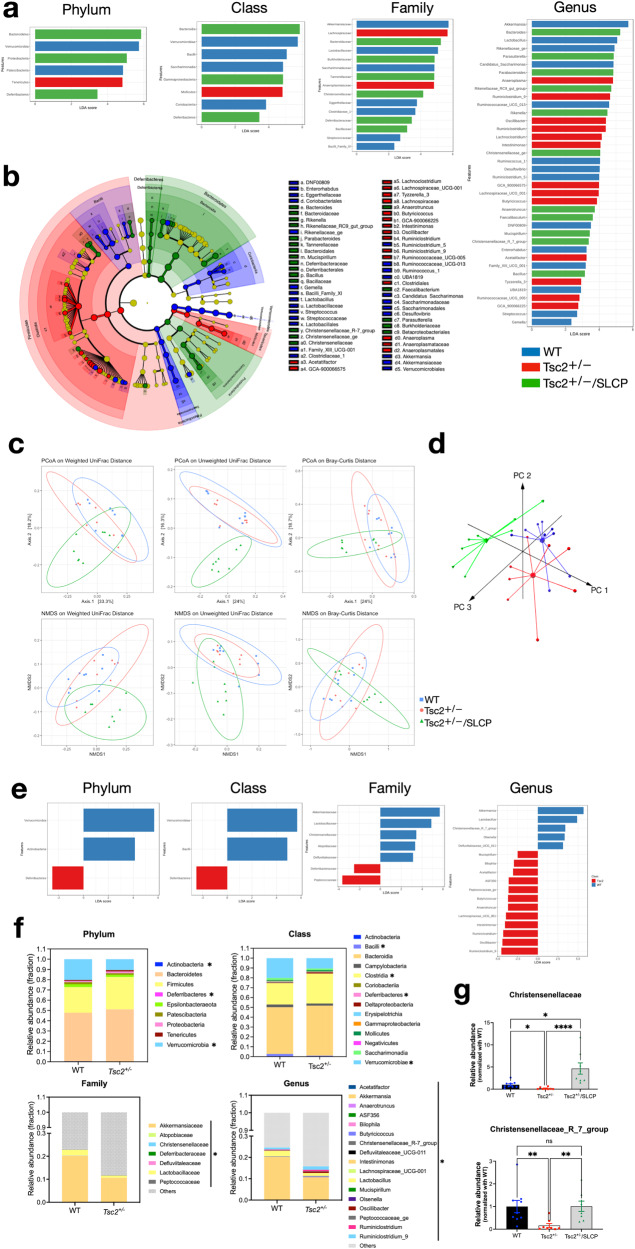
LEfSe gut microbiota analysis. **a** Histograms of the LDA scores of gut microbacterial taxa that are differentially abundant among the three groups. Taxa with LDA scores greater than 2 are listed according to the taxonomy as indicated. **b** A cladogram generated from LEfSe analysis depicts a taxonomic representation of statistical differences among the three groups. Each ring represents the next taxonomic level (from center to outer ring: phylum to genus). The size of each dot represents the relative abundance of the taxon. The color represents significant differences in the group when compared to other groups: blue: WT mice, red: *Tsc2*^*+/−*^ mice, green: *Tsc2*^*+/−*^/SLCP mice. Yellow dots are no significant differences among the three groups. α < 0.05, Kruskal-Wallis test **c** *Upper panel*. Beta-diversity graphs of PCoA on weighted or unweighted UniFrac and Bray-Curtis distance. *Lower panel*. Beta-diversity graphs of NMDS on weighted or unweighted UniFrac and Bray-Curtis distance. **d** 3D-plot of principle component analysis is shown. **e** Histograms of the LDA scores of gut microbacterial taxa that are differentially abundant between WT and *Tsc2*^*+/−*^ mice. Taxa with LDA scores greater than 2 are listed according to the taxonomy as indicated. **f** Histogram graphs that show the relative abundances of bacterial phyla, classes, families, and genera as indicated in each group. *p < 0.05, two-tailed Mann–Whitney test. **g** *Upper panel*: A bar graph that shows the relative abundance of the family Christensenellaceae in WT, *Tsc2*^*+/−*^, *Tsc2*^*+/−*^/SLCP mice. *Lower panel*: A bar graph that shows the relative abundance of the genus *Christensenellaceae_R_7_group* in WT, *Tsc2*^*+/−*^, *Tsc2*^*+/−*^/SLCP mice. WT (*N* = 9), *Tsc2*^*+/−*^ (*N* = 8), and *Tsc2*^*+/−*^/SLCP (*N* = 8) mice were assessed. Data represent the mean ± SEM. *p < 0.05, **p < 0.01, ****p < 0.0001, n.s. not significant, Kruskal Wallis, *post hoc* Uncorrected Dunn’s test.

To probe further into the mechanisms of SLCP treatment, we analyzed and compared the gut bacterial composition from the fecal DNA extracted from the three groups. We used linear discriminant analysis (LDA) effect size analysis (LEfSe) to explore the gut microbiota among the three groups. We computed the LDA score and listed the taxa, according to the phylum, class, family, and genus levels, that have a score greater than 2 (Fig. 5a). *Tsc2*^*+/−*^/SLCP group exhibited dramatically increased abundance in phyla Bacteroidetes, Proteobacteria, and Deferribacteres. At the class level, SLCP treatment increased the taxa Bacteroidia, Gammaproteobacteria, and Deferribacteres. Among bacterial families, *Bacteroidaceae, Burkholderiaceae, Tannerellaceae, Christensenellaceae, Deferribacteraceae*, and *Bacillaceae* were increased after the SLCP treatment. At the genus level, species in these genera were increased: *Bacteroides, Parasutterella, Parabacteroides, Rikenellaceae_RC9_gut_group, Rikenella, Christensenellaceae_ge, Anaerotruncus, Faecalibaculum, Mucispirillum, Christensenellaceae_R_7_group* and *Bacillus*. Then, derived from the LEfSe analysis, a cladogram showing the taxa with different abundances was generated (Fig. 5b). We next analyzed the beta diversity, which is the between-sample diversity, using principal coordinate analysis (PCoA) unweighted UniFrac and weighted UniFrac, variance adjusted weighted UniFrac, GUniFrac with alpha 0.5, and NMDS on Bray-Curtis distance (Fig. 5c, Table 2). The results of these analyses showed that the gut bacterial flora of WT and *Tsc2*^*+/−*^ clustered in separate groups, and the SLCP-treated group was predominately different from the other two groups. Principal component analysis (PCA) of operational taxonomic units (OTUs) abundance comparing the 3 groups demonstrated that each group has different clusters in 3 components (PC1, PC2, and PC3) (Fig. 5d).

**Table 2:** Beta-diversity profiling of gut microbiota and significance testing for wild-type, *Tsc2*^*+/−*^, and *Tsc2*^*+/−*^*/*SLCP groups.

Beta-diversity indices	Adonis P^a^	Betadisper P^b^
Unweighted UniFrac	0.000999	0.5085
Weighted UniFrac	0.01199	0.5674
Variance adjusted weighted UniFrac	0.000999	0.5425
GUniFrac with alpha 0.5	0.000999	0.9051
NMDS on Bray-Curtis distance	0.000999	0.3377

^a^Adonis P represents dissimilarity of composition among groups, *p* < 0.05 considered significant.

^b^Betadisper P was calculated to determine the homogeneity of dispersion.

### Gut microbiota composition differed between the WT and the Tsc2^+/−^ mice

To determine whether there is a genetic effect on gut microbiota composition, we sought to explore differences between WT and *Tsc2*^*+/−*^ mice by using the LEfSe method. When compared to the WT mice, the *Tsc2*^*+/−*^ mice displayed a decreased abundance of Verrucomicrobia and Actinobacteria, and an increased abundance of Deferribacteres (Fig. 5e). At the class level, the WT mice showed more abundant of Verrucomicrobiae and Bacilli, whereas *Tsc2*^*+/−*^ mice were more abundant of Deferribacteres. We also found 7 significant taxa at the family level, and 17 significant taxa at the genus level that differed between WT and *Tsc2*^*+/−*^ mice. In addition, we further confirmed this result using the relative abundance of the bacterial composition in each animal and found that some taxa significantly differed between WT and *Tsc2*^*+/−*^ mice (Fig. 5f). Based on these results, we proposed that the genetic deficiency of *Tsc2* may predispose the mice to gut dysbiosis. Interestingly, among these significant bacterial genera, the abundance of Christensenellaceae_R_7_group, which belongs to the family of Christensenellaceae, was significantly decreased in *Tsc2*^*+/−*^ mice, yet was reversed after the SLCP treatment (Fig. 5g). This particular species may represent an important gut microbiota biomarker for TSC and therapeutic target of SLCP.

### Correlations between gut microbiota abundance and DTI metrics

**Fig. 6:**
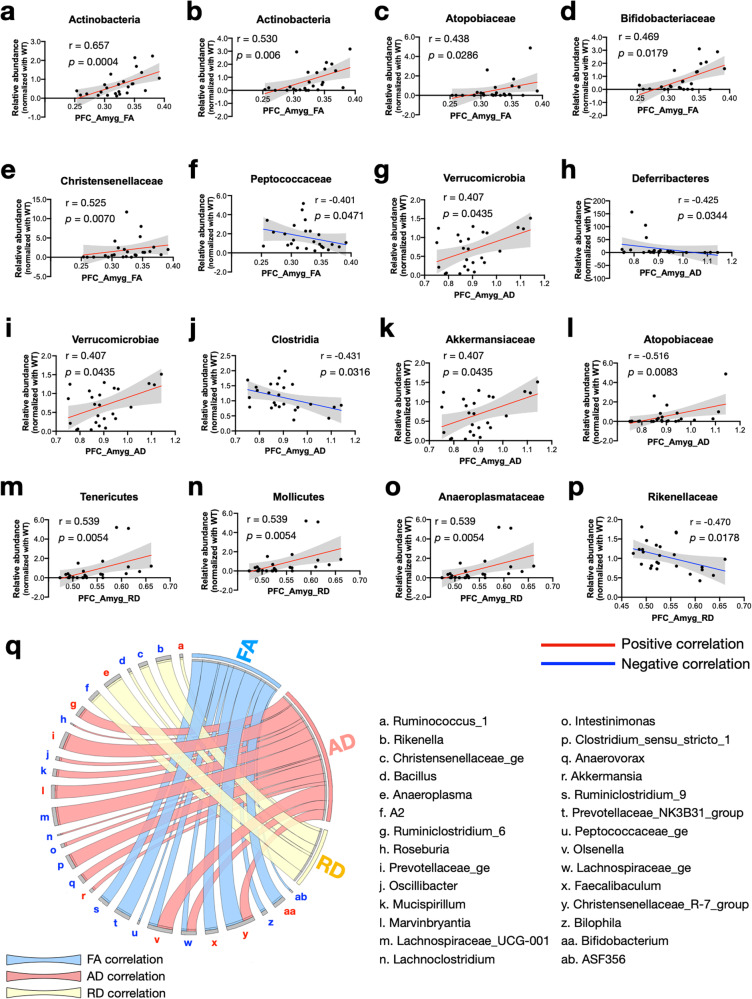
Correlation plots of DTI metrics and bacterial taxa abundances. **a–f** Scatter plots of the FA values of tracts between PFC and Amyg and the relative abundances of bacterial taxa in the phylum, class, and family with simple linear regression analysis. **g–l** Scatter plots of the AD values of tracts between PFC and Amyg and the relative abundances of bacterial taxa in the phylum, class, and family with simple linear regression analysis. **m–p** Scatter plots of the RD values of tracts between PFC and Amyg and the relative abundances of bacterial taxa in phylum, class, and family with simple linear regression analysis. **q** A circos plot shows the correlation between DTI metrics and the bacterial abundances at the genus level. The ribbon size represents the ranking of the r value in the correlation analyses. The color of each letter designated bacterial genus represents the positive (red) or negative (blue) correlation with the DTI metrics. WT (*N* = 9), *Tsc2*^*+/−*^ (N = 8), and *Tsc2*^*+/−*^/SLCP (*N* = 8) mice were assessed. The correlation coefficient Spearman (r) was computed set at a 95% confidence interval; the *p* value represents two-tailed. Significance set at α = 0.05.

To dissect more into the relationship between gut microbiota flora and brain connectivity, we generated correlation plots between the FA, AD, and RD values of the fiber bundles connecting the PFC-Amyg and the relative abundance of the gut bacteria for each animal. We pooled all the metrics, regardless of the genotype or the treatment, and plotted them against the relative abundance of each bacterial taxa. We found that the FA positively correlated with the relative abundance of the phylum Actinobacteria (Fig. 6a), the class Actinobacteria (Fig. 6b), the families *Atopobiaceae, Bifidobacteriaceae Christensenellaceae* (Fig. 6c–e); and it is inversely correlated with the family *Peptococcaceae* (Fig. 6f). On the other hand, AD showed positive correlations with the phylum Verrucomicrobia and a negative correlation with the phylum Deferribacteres (Fig. 6g, h). At the class level, AD also showed a positive correlation with Verrucomicrobiae, while showed a negative correlation with Clostridia and Deferribacteres (Fig. 6i, j). In addition, at the family level, AD had a positive relationship with *Akkermansiaceae* and *Atopobiaceae*, whereas an inverse relationship with *Deferribacteraceae* (Fig. 6k, l). RD value showed a positive correlation with the phylum Tenericutes, the class Mollicutes, the family *Anaeroplasmataceae*, and a negative correlation with the family *Rikenellaceae* (Fig. 6m–p).

We further analyzed the relationships between DTI metrics versus different bacterial species at the genus level. 10 genera showed significant correlations with FA, 6 of them were positively correlated, and 4 of them were inversely correlated. For AD value, a total of 15 genera showed correlations, where 7 of them were positive correlations, and 8 of them were negative correlations. Interestingly, we found that both FA and AD values showed positive correlations with Christensenellaceae_R_7_group, Olsenella, and Lachnospiraceae_ge. For RD value, on the other hand, only 2 genera were positively correlated, and 4 of them were inversely correlated (Fig. 6q, Table 3).

**Table 3:** Significant correlations between DTI metrics and gut bacterial taxa at the genus level.

DTI metrics	Genus	Spearman r-value	*p* value	Correlation
FA	Christensenellaceae_R-7_group	0.6469	0.0005	Positive
	Bifidobacterium	0.4694	0.0179	
	Faecalibaculum	0.4671	0.0186	
	Olsenella	0.4379	0.0286	
	ASF356	−0.3992	0.048	Negative
	Peptococcaceae_ge	−0.4008	0.0471	
	Bilophila	−0.4188	0.0372	
	Lachnospiraceae_ge	−0.4238	0.0347	
	Ruminiclostridium_9	−0.4531	0.0229	
	Prevotellaceae_NK3B31_group	−0.4996	0.011	
AD	Prevotellaceae_ge	0.5546	0.004	Positive
	Olsenella	0.5157	0.0083	
	Clostridium_sensu_stricto_1	0.4864	0.0137	
	Marvinbryantia	0.4844	0.0141	
	Christensenellaceae_R-7_group	0.4600	0.0207	
	Ruminiclostridium_6	0.4574	0.0215	
	Akkermansia	0.4069	0.0435	
	Lachnoclostridium	−0.3977	0.049	Negative
	Roseburia	−0.3977	0.049	
	Intestinimonas	−0.4115	0.041	
	Oscillibacter	−0.4115	0.041	
	Mucispirillum	−0.4246	0.0344	
	Anaerovorax	−0.4281	0.0328	
	Lachnospiraceae_ge	−0.4377	0.0287	
	Lachnospiraceae_UCG-001	−0.6721	0.0002	
RD	Anaeroplasma	0.539	0.0054	Positive
	Ruminococcus_1	0.4008	0.0471	
	Bacillus	−0.4021	0.0463	Negative
	Christensenellaceae_ge	−0.4052	0.0445	
	A2	−0.4428	0.0267	
	Rikenella	−0.4492	0.0243	

## Discussion

Our study provides evidence that SLCP treatment improved the abnormal social behaviors in the *Tsc2*^*+/−*^ mice, strengthened the connectivity between several key brain regions involved in cognition and sociability through increasing MBP and PLP expression, and coinciding with alterations in the gut microbial composition. Herein, we proposed that the SLCP treatment, which has long been known to possess anti-inflammatory, anti-oxidant, and mTOR inhibitory functions, regulated astroglial and oligodendroglial functions, and may exert additional beneficial effects through shaping the intestine bacterial composition in the *Tsc2*^*+/−*^ mouse model.

In this present study, we demonstrated that psychiatric disease-related behaviors in the *Tsc2*^*+/−*^ mice, including abnormal cognitive ability and sociability, resulted from disrupted brain circuitries. FA and AD reductions of anterior forceps in *Tsc2*^*+/−*^ mice corresponded to a shifted distribution of axon sizes (Fig. 4e). The frequency of axons with diameters 0.4–0.5 μm has increased from 5.28% in WT mice to 10.14% in *Tsc2*^*+/−*^ mice (about a two-fold increase), while the frequency of axons with diameters 1.0–1.1 μm has dramatically decreased from 9.62% in WT mice to 3.22% in *Tsc2*^*+/−*^ mice (about a three-fold decrease). Both FA and AD values have been used to characterize axonal changes [64]. FA represents the degree of water diffusion direction in fiber tracts, whereas AD indicates the amount of water diffusion parallel to the tracts in a voxel, which is more sensitive to changes in axonal tract integrity (i.e., axonal injury or reduced axonal caliber may give rise to a reduced AD) [65, 66]. Since the properties of WM fiber geometry, such as axon diameter or density, have been suggested to be reflected by FA and AD values [19, 67, 68], these differences in axons shown by TEM analysis may explain the reduced FA and AD values in the DTI results.

Furthermore, FA has been suggested to be strongly associated with myelination (see review papers [19, 67]). One of the examples is a study conducted by Sampaio-Baptista et al. [69] The WM of rats that were trained in a motoric task showed an increase in FA when compared to the control group. Subsequent immunohistological analysis revealed an increase in myelin staining, suggesting a link between FA and myelin. Furthermore, a study carried out by Peters et al. [10] also revealed the correlation between FA and myelination in the WM of TSC patients. Consistent with these studies, this current study also demonstrated that myelin breakdown in *Tsc2*^*+/−*^ mice may contribute to the reduced FA value. And the *Tsc2*^*+/−*^/SLCP group showed increased MBP expression (Fig. 4f, g), corresponding to an elevation in the FA (Fig. 3e).

By comparison, RD has been suggested to be more sensitive to myelin changes, since it measures the amount of water diffusion perpendicular to the axonal tract [19]. Therefore, as shown in many studies, an increment in RD means increased perpendicular water movement in the fiber tracts, which may indicate damage or aberrant structures of the myelin sheaths [19, 21, 66, 70]. Likewise, we demonstrated that SLCP treatment enhanced the structure of myelin sheaths (Fig. 4b) through increasing MBP expression (Fig. 4f, g), and RD was decreased after the treatment (Fig. 3g). Our results, in line with other studies, also confirmed the direct relationship between MBP expression and RD metrics.

However, we did not detect changes in RD or MBP and PLP expression in *Tsc2*^*+/−*^ mice, even though we observed an increased frequency of myelin breakdown in this mouse model (Fig. 4d). This seemingly contradictory result is consistent with other research teams [71, 72]. The integrity of the myelin sheath is rather complicated and is proposed to be affected by several processes, which include the differentiation and maturation of OLs, myelin sheath formation and lamination, and axon-glia interaction [11, 73, 74]. However, the underlying mechanism of myelination in CNS remains elusive. Past research indicated that the TSC-associated myelin impairments may be caused by the maturation of OLs at early stages of life, since increased immature OLs (indicated by increased NG2-positive cells) were consistently detected in the TSC rodents, the human patient iPSC model, and as well as in TSC patient-derived primary glial cells [72, 75, 76]. Thus, we proposed that abnormal oligodendroglial differentiation and maturation may have caused the myelin sheath defects observed in *Tsc2*^*+/−*^ mice.

Curcumin has been known to have great therapeutic efficacy in treating neurodegenerative and neurological diseases because of its anti-oxidant [77, 78], anti-inflammatory [79–81], and mTOR-inhibiting properties [82, 83]. As shown in literature, mTOR complex 1 (mTORC1) is indispensable during the myelination process, which includes differentiation of OLs from oligodendrocyte precursor cells, lipid and myelin protein synthesis, and myelin sheath production (see the review paper [84]). Using different cell type-specific knockout TSC mouse models, several groups showed that inhibiting mTORC1 using rapamycin has successfully rescued neuronal, oligodendroglial, astroglial, and microglial defects in the TSC rodent models [7, 85–87]. Moreover, past research has revealed that curcumin is able to increase MBP expression, promoting myelin sheath formation and establishing remyelination in peripheral nerves and corpus callosum in the central nervous system [88, 89].

In our previous studies, we have also confirmed that SLCP is able to refine the overactivation of mTOR in *Tsc2*^*+/−*^ mice, and induce changes in astroglia and myelination patterns (but not in microglia), thereby improving the cognitive and anxiety-like performance in the novel object recognition and open field behavioral tests, respectively [39, 90]. Based on these results, we propose that one of the molecular mechanisms by which SLCP mitigated the abnormal myelination and enhanced brain connectivity in the *Tsc2*^*+/−*^ mouse model is its mechanism of action on mTORC1 and downstream pathways.

In addition to mTOR-inhibiting activity, curcumin has been suggested by recent findings to have additional pharmacological roles through regulating gut microbiota to exert its neuroprotective functions [63, 91]. The administration of curcumin alters the microbial richness and diversity in mice, as it tends to increase the beneficial bacteria, while decreasing the abundance of bacterial taxa that are often linked to metabolic and inflammatory diseases [91–93]. In this present study, we showed that the curcumin diet may greatly change the microbial composition in *Tsc2*^*+/−*^ mice and favor the growth of certain bacterial strains that may promote the myelination process.

Gut microbiota affects the brain by releasing substances including neurotransmitters, metabolites such as amino acids and short-chain fatty acids (SCFAs), and modulators involved in immune system pathways [94]. Recently, there has been increasing evidence that gut microbiota-derived metabolites are necessary for the myelination process [33–35, 95] and maintaining an intact WM structure in a diet-dependent manner [96]. For example, supplementing an antibiotics-induced dysbiosis mouse model with bacterial SCFA butyrate is able to restore the PFC myelination, through normalizing the myelin gene expression in the PFC [35]. In this current study, we identified an increased abundance of some butyrate-producing bacteria in the SLCP-treated group, i.e., *Faecalibacterium, Bacteroidetes*, and *Proteobacteria* [97]. This observation may provide additional evidence to support the link between SCFA butyrate and myelination, as well as the possible mechanisms by which SLCP mitigates the myelination.

In recent years, GBA has gained much attention particularly how it exerts impacts on neuropsychiatric disorders like ASD. Several human and animal studies have reported aberrant gut microbiota composition in ASD individuals and preclinical models [98]. The influence of human genetics on ASD has also been studied for many years. Nowadays, it is well-accepted that both environmental and genetic factors are equally important for the development of ASD [99]. Here we showed that the genetic deficiency of *Tsc2* has been attributed to a different gut microbiome, predisposing the animal to atypical social behaviors. We observed that the relative abundances of 5 genera were decreased and 12 genera were increased in *Tsc2*^*+/−*^ mice (Fig. 5e). Among these differentially colonized bacteria, *Akkermansia*, *Bilophila*, and *Lactobacillus* are closely related to dysbiosis in ASD clinical cases and preclinical models of ASD [98].

Interestingly, among these significant differed microbial members, the abundance of *Christensenellaceae_R_7_group*, which belongs to the family of Christensenellaceae, was significantly decreased in *Tsc2*^*+/−*^ mice, and was reversed after the SLCP treatment. The *Christensenellaceae* family has been consistently reported to be the most highly heritable taxon in both mouse and human gut microbiomes [100, 101]. This family of microbiota may represent an important microbial biomarker, of which the colonization is highly dependent with the host genome. Indeed, we showed that the deficiency of *Tsc2* correlated with the low abundance of *Christensenellaceae*. As the low abundance of *Christensenellaceae* is highly associated with poor metabolic health and obesity in several human studies [100, 101], we proposed that this bacteria is also highly related with TSC.

The metabolic dysfunction has been suggested in TSC, based on the hamartin/tuberin involvement in cellular metabolic pathways and some case report studies [102–104]. However, whether or not the abundance of this bacterial family, or other bacterial taxa mentioned above, represents a specific feature of the TSC, a longitudinal monitoring of gut microbiome at different stages of *Tsc2*^*+/−*^ mice is required to clarify this matter. As gut bacteria are susceptible to environmental cues, by comparing gut microbiota profiling at different stages between WT and *Tsc2*^*+/−*^ mice will help us to find the specific bacterial taxa which could represent as the unique gut microbial profiling of this disease.

In the present study, we found that the SLCP treatment increased RD metrics and MBP expression and restored the g-ratio of myelinated axons in the WM region anterior forceps. At the same time, when compared to the treatment-naïve groups (WT and *Tsc2*^*+/−*^ mice), the SLCP-treated group showed an increase in the relative abundance of the phylum Bacteroidetes and a decrease in the phylum Tenericutes. Indeed, the abundance of Tenericutes showed a positive correlation when it was plotted against the RD value (Fig. 6m), suggesting that Tenericutes may play a role in myelination. This hypothesis may be further supported by a previous study, which described that members of Tenericutes are enriched in a mouse model of multiple sclerosis, a known neurodegenerative disease characterized by the destruction of myelin sheaths [105]. At the genus level, RD positively correlated with *Ruminococcus_1* and *Anaeroplasma*, suggesting these bacteria may have a detrimental effect on myelination. On the other hand, RD negatively correlated with *Rikenella, Christensenellaceae_ge, Bacillus*, and *A2*, indicating these bacteria may have a beneficial effect on myelination. However, further studies are required to further explore the roles of these bacteria and myelination.

The fact that the neurodevelopment, as well as myelin and white matter maturation during postnatal development, coincides with the colonization of gut microbiota in childhood and adolescence has prompted researchers to seek the possibility of treating neuropsychiatric disorders through improving gut microenvironment at these critical time windows. Our study further supports the fact that the maintenance of a healthy gastrointestinal tract is crucial for preventing behavioral and neuropsychiatric disorders [32].

## Conclusion

Our study utilized a non-invasive, tract-based DTI analysis to detect impaired structural connectivity within brain regions, PFC, NAc, HyTh, and Amyg, which are associated with abnormal social behavior in *Tsc2*^*+/−*^ *mice*. The DTI metrics FA and AD reflected the connectivity and axonal alterations, respectively, in *Tsc2*^*+/−*^ mice. Further, we performed gut microbiome analysis and found that deficiency of *the Tsc2* gene causes different gut microenvironments, which may be responsible for decreased connectivity and sociability. Furthermore, we demonstrated that dietary treatment of SLCP is able to reverse the sociability deficits and the FA values. In addition, the treatment increases the expression level of MBP and PLP, and lowers the RD value. The gut microbiome analysis showed that the bacterial composition tremendously differed after the treatment. We found that the abundance of certain bacteria taxa was greatly increased. These changes corresponded to increased myelination and white matter plasticity, contributing to improved sociability in *Tsc2*^*+/−*^ mice.

### Supplementary information

Supplementary

## Data Availability

The data that support the findings of this study are available from the corresponding author upon reasonable request.
