# Supplementary material for: Amelioration of the brain structural connectivity is accompanied with changes of gut microbiota in a tuberous sclerosis complex mouse model
Source: Transl Psychiatry. 2024 Jan 31;14:68. doi: 10.1038/s41398-024-02752-y (PMC10830571; doi:10.1038/s41398-024-02752-y)
Supplement: Supplementary file 1 — Supplementary [file 41398_2024_2752_MOESM1_ESM.docx]

**Supplementary Information**

**Amelioration of the Structural Connectivity in a Tuberous Sclerosis Complex Mouse Model by Regulating Glial Functions via Gut Microbiota**

Christine Chin-jung Hsieh^1#^, Yu-Chun Lo^2,3#^, Hsin-Hui Wang^2^, Hsin-Ying Shen^1^, You-Yin Chen^2,4^*, Yi-Chao Lee^2,3,5^*

^1^Biomedical Translation Research Center, Academia Sinica, Taipei, Taiwan

^2^Ph.D. Program in Medical Neuroscience, College of Medical Science and Technology, Taipei Medical University, Taipei, Taiwan

^3^Neuroscience Research Center, Taipei Medical University, Taipei, Taiwan

^4^Department of Biomedical Engineering, National Yang Ming Chiao Tung University, Taipei, Taiwan

^5^International Master Program in Medical Neuroscience, College of Medical Science and Technology, Taipei Medical University, Taipei, Taiwan

#These authors contribute equally to this work.

*Correspondence should be addressed to:

Professor You-Yin Chen, Department of Biomedical Engineering, National Yang Ming Chiao Tung University, No.155, Sec.2, Linong St., Taipei, Taiwan 11221

E-mail: irradiance@so-net.net.tw

Professor Yi-Chao Lee, Ph.D. Program in Medical Neuroscience, College of Medical Science and Technology, Taipei Medical University, No. 250, Wuxing St., Taipei, Taiwan 11031

E-mail: yclee@tmu.edu.tw

**Running head:** Structural connectivity and alterations in gut microbiota in a TSC mouse model


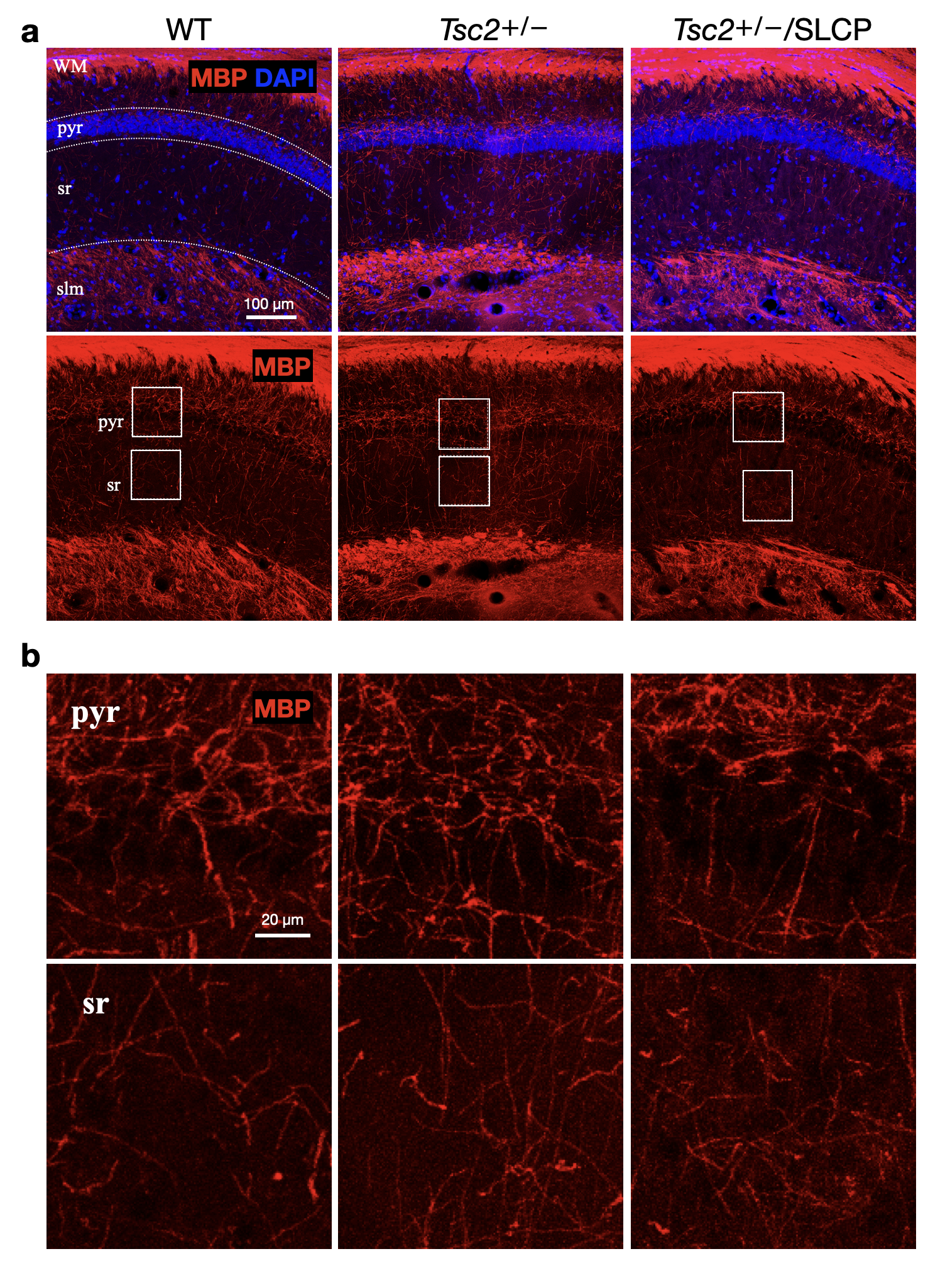


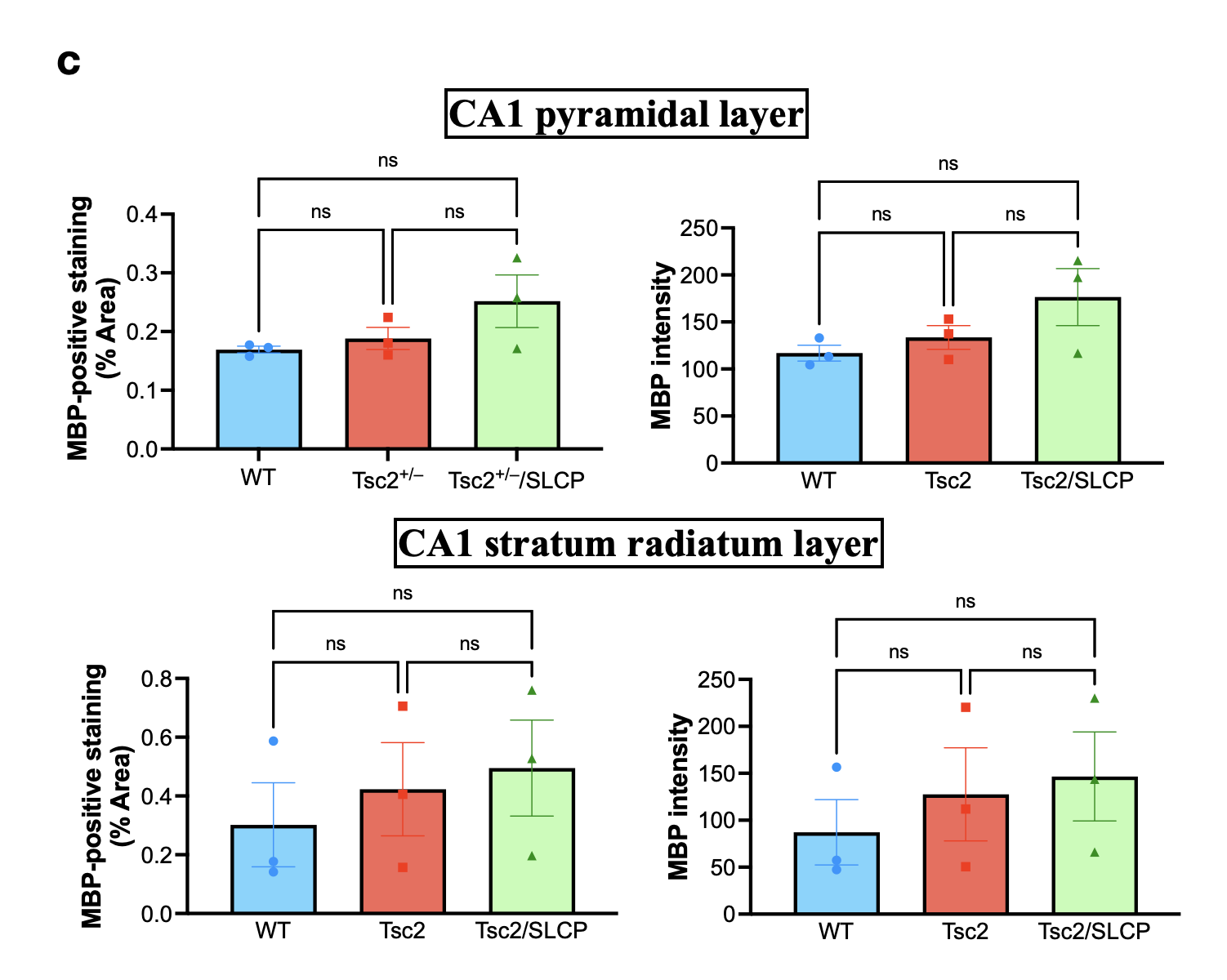


**Figure S1**. **Fluorescent immunostaining of myelin basic protein (MBP) on the hippocampal CA1 region.**

**a** A representative fluorescence immunostaining image from each group is shown for visualizing the immunoreactivity of MBP staining in the hippocampal CA1 region. **b** Magnified images of the boxed regions in the above images. Since the patterns and immunointensity of CA1-pyr and CA1-sr are different, we calculated them separately. **c** The quantitative bar graph shows the MBP-positive staining area (%) for the three groups in CA1-pyr and CA1-sr. Abbreviations: MBP: myelin basic protein; CA1: cornu ammonis 1; pyr: pyramidal layer; sr: stratum radiatum layer. WT (N = 9), *Tsc2^+/−^* (N = 8), and *Tsc2^+/−^*/SLCP (N = 8) mice were assessed. Data represent the mean ± SEM. **p* < 0.05, ***p* < 0.01, n.s. not significant, One-way ANOVA test, *post hoc* Tukey’s test.
